# 25 Lat Działalności Zespołu Interdyscyplinarnego Do Spraw Wad płodu Przy Instytucie Matki i Dziecka- Od Poradnictwa Do Etyki Klinicznej

**DOI:** 10.34763/devperiodmed.20192304.246252

**Published:** 2021-01-29

**Authors:** Magdalena Rutkowska, Sławomir Szczepaniak, Tomasz Mikołaj Maciejewski, Renata Jaczyńska, Ewa Helwich, Marzanna Reśko-Zachara

**Affiliations:** 1 Klinika Neonatologii i Intensywnej Terapii Noworodka, Instytut Matki i Dziecka, Warszawa, Polska; 2 Papieski Wydział Teologiczny, Collegium Joanneum, Warszawa, Polska; 3 Klinika Położnictwa i Ginekologii, Instytut Matki i Dziecka, Warszawa, Polska

**Keywords:** perinatalna opieka paliatywna, wady wrodzone, zespół interdyscyplinarny, etyka kliniczna, perinatal palliative care, birth defects, interdisciplinary teams, clinical ethics

## Abstract

Perinatologia jest bardzo prężnie rozwijającą się dziedziną, w której rozwój techniki w ostatnich dziesięcioleciach umożliwił coraz wcześniejsze rozpoznawanie wad wrodzonych, w tym o przebiegu letalnym, czy zespołów genetycznie uwarunkowanych. W wielu sytuacjach klinicznych praca jednoosobowa jest już niemożliwa, zarówno w postawieniu ostatecznej diagnozy, zaplanowaniu leczenia czy przewidzenia rokowania dla chorego płodu/noworodka. Istnieje konieczność pracy zespołowej, która umożliwia należytą opiekę nad tak chorym dzieckiem już w okresie płodowym, a nie tylko na sali porodowej czy OITN.

W pracy omówiono podstawy etyczne tworzenia zespołów interdyscyplinarnych oraz biorąc jako przykład pracujący od 25 lat Zespół Interdyscyplinarny do spraw Wad Płodu w Instytucie Matki i Dziecka w Warszawie pokazano jego działanie w praktyce.

W artykule podano wypracowane zasady pracy Zespołów Interdyscyplinarnych oraz praktyczne wskazówki dotyczące kontaktu lekarzy informujących o sytuacji klinicznej dziecka z jego rodzicami i wspólnie zaplanowanie opieki nad nim po urodzeniu.

## Abstract (en)

Perinatology is a dynamically developing field of medicine. The progress of technology in recent decades has made it possible to recognize birth defects very early, inc/uding those which are lethal or genetically determined. In many clinical situations, it is no longer possible to work a/one to reach a definitive diagnosis, plan treatment or predict the clinical course of the affected fetus/newborn. There is a need for teamwork, which ensures the proper, early care starting in the fetal period, not only in the delivery room or NICU.

This paper discusses the ethical foundations of creating interdisciplinary teams, taking as an example the Interdisciplinary Team for Fetal Malformation at the Institute of Mother and Child in Warsaw, which has been active for 25 years, and presents how it works in practice.

On the basis of the clinical cases that are examined, practical guidelines were formulated for both the work of medical teams and the way parents are informed about the clinical situation of their child and about sharing the care after the birth of the child. A document was also proposed with information on prenatal diagnosis, decisions made by the interdisciplinary team regarding the mother and child, as well as the conclusions resulting from discussions with parents.

## Dlaczego zespół interdyscyplinarny?

Okres prenatalny to czas najbardziej spektakularnego rozwoju: od mikroskopijnej zygoty do około pięćdziesięciocentymetrowego noworodka. Wzrastanie to zależy od wielu czynników: genetycznych, środowiskowych i hormonalnych. Wszystkie one mogą podlegać pewnym ograniczeniom lub wprowadzać zmiany w ten jakże dynamicznie postępujący i złożony proces. Trudno się zatem dziwić, że mogą tu występować błędy – błędy, które koryguje sama natura. Ocenia się, że od 50 do 80% wczesnych poronień jest spowodowanych poważną wadą rozwojową płodu. Jednak natura i tu dopuszcza pewne wyjątki. U 2-3% żywo urodzonych noworodków występują izolowane duże wady wrodzone, zaś u około 0,7% stwierdza się mnogie wady wrodzone. Wady te stanowią przyczynę ok. 20% zgonów okołoporodowych [1]. Niezależnie od zastosowanego leczenia, mogą one stać się przyczyną śmierci w okresie noworodkowym lub niemowlęcym.

Problem wad i chorób rzadkich nabiera szczególnej wagi, jeśli zgodzimy się z tezą Luca Ferry, że żyjemy w czasach „rewolucji miłości” [2]. Na podobieństwo „rewolucji komunistycznej” lub „rewolucji obyczajowej”, Luc Ferry „rewolucją miłości” nazywa czasy, w których człowiek gotów jest „poświęcić się” dla tych, których kocha, a w szczególności dla swoich dzieci. Według tego filozofa, obecnie człowiek nie ma już zamiaru oddawać swojego życia za Boga, honor, wolność czy ojczyznę. Dziś, w epoce końca wielkich ideałów, triumfu ciasno rozumianego komfortu i wszechpanującej konsumpcji, cóż może być świętszego nad własne dziecko, nad zobowiązanie miłości podjęte w stosunku do własnej rodziny? Ta właśnie miłość znajdująca się w centrum naszego istnienia stanowi odpowiedź na podstawowe pytanie o sens życia. W przypadku gdy życie dziecka jest zagrożone rzadką chorobą, wadą wrodzoną, cały ten misternie utkany humanizm zaczyna się walić. W imię „rewolucji miłości” człowiek gotów jest podjąć heroiczną walkę, krzycząc: „Moje dziecko, moja walka!” I tej walce gotów jest poświęcić wszystko. Dysponuje przy tym ogromnym potencjałem technicznym i farmakologicznym, pozwalającym na dowolnie długie podtrzymywanie czynności życiowych, ale czy takie postępowanie ma jakikolwiek sens? Czy w chwili, gdy walka wydaje się być z góry przegrana, postawa taka nie będzie równoznaczna z tym, co starożytni nazywali *hubris*, to znaczy brakiem umiaru? Potrzeba tu rozwagi. Potrzeba kogoś, kto pokaże teren walki i granice ludzkich możliwości. *Hubris* nie dotyczy jednak wyłącznie rodziców heroicznie walczących o życie swoich dzieci. Może ona łatwo zagnieździć się na każdym poziomie medycznego działania i zawładnąć również osobą samego lekarza, który uwiedziony sukcesami medycyny, traci z oczu granice swoich własnych kompetencji. Może też poruszony współczuciem zacząć sobie tłumaczyć konieczność przekroczenia pewnych granic, wyznając przy tym wiarę we wszechmoc samej medycyny. Zatraca się on wtedy i zapomina, jaka jest rzeczywista rola lekarza.

Dziś słowo „lekarz” nie znaczy wiele – to ten, który odmierza lekarstwa. W dawniejszych czasach używano słowa „medyk”, które etymologicznie pochodzi od łacińskiego *medeor (medeor alicui* – leczę kogoś). W językach indoeuropejskich partykuła *med-* pojawia się, gdy mowa jest o myśleniu (np. medytować, mędrkować), opiece (np. medium w znaczeniu pośrednika, opiekuna), rządzeniu (np. mediator w sensie arbiter, sędzia) oraz o mierze (np. mediować w sensie odnaleźć słuszną miarę, ile należy wziąć z każdego, by osiągnąć zamierzony cel). Właśnie to wyrażenie miary, nie w sensie mierzenia, ale wyznaczania właściwej miary, czyli „umiarkowania” stanowi podstawę rozumienia słowa „medyk”. Oto zadanie, które starożytni przypisywali medycynie: przywrócenie właściwej miary gwarantującej odbudowanie porządku. Lekarz może tu być postawiony na równi z *Zeùs medéōn*, to znaczy Zeusem Moderatorem wyprowadzającym świat z chaosu i przeprowadzającym go do kosmosu, czyli tym, który światu nadaje porządek. Aby wykonać to zadanie medyk – na podobieństwo Zeusa Moderatora – posiada *mēdea eidōs* czyli miarę rzeczy, dzięki której może on zarządzać naturą. Wie on, jaka miara przynależy wszelkim rzeczom, czego jest za dużo, a czego za mało. Zarządzać naturą nie znaczy ją zastępować, byłoby to bowiem przekroczeniem miary, czyli wykroczeniem, po grecku – *hubris*.

Jedynym lekarstwem na tak rozumiane *hubris* rodziców lub samego lekarza – zgodnie z tym, co proponuje Arystoteles – jest *phronesis*, czyli mądrość praktyczna, swoista przenikliwość, pozwalająca na odnalezienie właściwej miary [3]. *Phronesis* jest córką szeroko pojętego doświadczenia *(emperia)*. Nie ogranicza się ona wyłącznie do własnego doświadczenie, ale zakłada doświadczenie jak najszerszego grona osób, które znają określone zagadnienie. Posiąść ją może tylko ten, kto potrafi czerpać z doświadczenia kogoś, kto dysponuje znacznie większym doświadczeniem i kogo możemy nazwać *phronimos*. Człowiek taki – jak mówi Arystoteles – „w sposób piękny rozważa nad sobą”. Rozważać nad sobą w sposób piękny znaczy: umieć rozróżnić między tym, co dobre i użyteczne dla siebie samego *(auto)*, a tym, co dobre i użyteczne dla człowieka jakim jestem, a zatem nad tym, czego każdy człowiek poszukuje. Kto może być dla nas owym powszechnie uznanym autorytetem etyko-medycznym, owym *pronimos* potrafiącym odróżnić to, co jest dobre dla rodzica (np. walczyć ze wszystkich sił o życie dziecka), lekarza (np. nie brać na własne sumienie śmierci niewinnego dziecka), a tym, co dobre dla nas jako niosących człowieczeństwo (np. pogodzenie się z faktem, że życie każdego człowieka nawet najmniejszego naznaczone jest cieniem śmierci)? Potrzebujemy wsparcia.

Często proponuje się, aby takim wsparciem były tzw. „Komitety Etyczne”. Zasiadają w nich zwykle obok lekarzy, eksperci, specjaliści od prawa, wysokiej rangi urzędnicy, którzy tworzą prawo, łub zgodnie z istniejącymi stanem prawnym rozsądzają, na ile proponowana procedura medyczna jest „etyczna” (czytaj: legalna). Często nie znają oni materii problemu, a ich ocena jest czysto formalna, jeśli nie polityczno-ideologiczna.

Jeśli chcemy wprowadzić do medycyny postępowania zgodne z mądrością praktyczną, potrzebujemy nie tyle „Komitetów Etycznych”, co raczej „Zespołów interdyscyplinarnych”. Tylko dzięki spotkaniu i dyskusji specjalistów z różnych dziedzin jesteśmy w stanie odnaleźć mądrość praktyczną i nadać naszemu postępowaniu medycznemu charakter racjonalny, to znaczy ukazujący człowieka w całej jego kompleksowości i spójności. Zespoły interdyscyplinarne mogą pełnić dziś funkcję owego mistrza, *phronimos*, który posiadł ogromne doświadczenie i który potrafi odróżnić własne cele: spokój sumienia, ślepą wiarę w procedury medyczne lub cud i nagłą zmianę sytuacji, niechęć do podejmowania decyzji, współczucie dla rodziców dziecka, uległość wobec naznaczonych chwilowym uczuciem decyzji rodziców, od tego, co jest prawdziwym dobrem naszego pacjenta, nawet tego najmniejszego i to ze względu na jego człowieczeństwo.

## Zespół interdyscyplinarny –działanie w praktyce

Przykładem takim może być istniejący już od 25 lat Zespół Interdyscyplinarny do spraw Wad Płodu w Instytucie Matki i Dziecka [4]. W skład zespołu wchodzą lekarze różnych specjalności z dużym doświadczeniem klinicznym, dzięki czemu mogą oni dokonać rzetelnej analizy patologii zaobserwowanej u płodu. Mogą też oni przedstawić rokowania co do dalszego rozwoju płodu a następnie dziecka po urodzeniu. Każdy przypadek jest rozpatrywany jednostkowo. Podstawą do zwołania posiedzenia Zespołu jest stwierdzenie przez położnika/ /perinatologa wady letalnej, jak również rozpoznania ciężkiej wady rozwojowej lub zespołu wad o trudnym do przewidzenia rokowaniu. Zespół może być zwołany przez każdego z jego członków. Często odbywa się na prośbę genetyka po stwierdzeniu u płodu nieprawidłowości genetycznych. Opinie specjalistów z danych dziedzin medycyny mają pomóc rodzicom w zrozumieniu choroby dziecka, oraz przygotowaniu do nowej, trudnej sytuacji po jego urodzeniu, a lekarzom do postępowania z noworodkiem w pierwszych chwilach jego życia.

W sytuacji, gdy rozpoznanie wady jest pewne a rokowanie znane, matka dziecka powinna być objęta natychmiastową opieką położniczą, neonatologiczną i psychologiczną przez ośrodek mający doświadczenie w prowadzeniu takich ciąż (najczęściej III stopnia referencji lub hospicjum perinatalne mające bezpośredni kontakt ze szpitalem, gdzie odbędzie się poród). W przypadku wady spełniającej warunki ustawy o planowaniu rodziny, ochronie płodu ludzkiego i warunkach dopuszczalności przerwania ciąży, pacjentka ma prawo złożyć pisemny wniosek z prośbą o wcześniejsze zakończenie ciąży [5]. Matce/rodzicom powinna być również zaproponowana opieka paliatywna dla ich dziecka i omówiony jej przebieg biorąc pod uwagę różne sytuacje w okresie ciąży (w tym możliwość zgonu w/macicznego) i sytuacji po urodzeniu.

Zupełnie inne są okoliczności, kiedy nie można od razu postawić ostatecznego rozpoznania i wymaga ono wykonania dodatkowych badań diagnostycznych (w tym genetycznych), obrazowych (np. rezonans magnetyczny płodu) i specjalistycznych konsultacji (np. kardiologicznych, kardiochirurgicznych, neurologicznych czy chirurgicznych). W takiej sytuacji najlepszą możliwością jest tworzenie Zespołów Interdyscyplinarnych w szpitalach wysokospecjalistycznych, które nie tylko ułatwiają postawienie rozpoznania, ale również skracają czas oczekiwania na rzetelną informację w tych kluczowych dla życia dziecka chwilach.

Oto trzy przykłady takiego właśnie postępowania podjętego przez Zespół Interdyscyplinarny do spraw Wad Płodu w Instytucie Matki i Dziecka w Warszawie.

### Przykład 1

Pacjentka K. L-N: w trakcie diagnostyki prenatalnej stwierdzono u płodu wadę letalną pod postacią Zespołu Edwardsa wraz z niewielką przepukliną pępowinową. Po dokonaniu analizy wady i przeprowadzeniu dyskusji wewnątrz Zespołu, wybrani członkowie (położnik, chirurg, neonatolog) spotkali się z matką, by omówić możliwości kontynuowania ciąży i postępowania z dzieckiem po urodzeniu. Przedstawiono mamie 4 sytuacje, jakie mogą wystąpić w trakcie ciąży i po urodzeniu dziecka:

1) Samoistne obumarcie wewnątrzmaciczne płodu.

2) Poród przedwczesny – ze względu na wadę letalną nie podejmowanie resuscytacji dziecka po urodzeniu i objęcie go opieką paliatywną (leczenie przeciwbólowe, jeżeli dziecko

będzie tego wymagało). Poproszono przy tym rodziców, aby zastanowili się, czy chcieliby ochrzcić dziecko zaraz po urodzeniu, jakim imieniem nazwać oraz jakie pamiątki chcieliby zgromadzić (zdjęcie, odbicie rączki, stopki), czyjej obecności życzyliby sobie po urodzeniu dziecka?

3) Poród o czasie z zabiegiem chirurgicznym – poród siłami natury (ze względu na wadę letalną). W przypadku stwierdzenia u dziecka po urodzeniu *uszkodzenia* worka przepukliny pępowinowej przeprowadzenie zabiegu operacyjnego w celu zamknięcia przepukliny i przygotowania dziecka do opieki hospicyjnej w warunkach domowych.

4) Poród o czasie bez zabiegu chirurgicznego – poród siłami natury (ze względu na wadę letalną). W przypadku stwierdzenia u dziecka po urodzeniu *zachowanego* worka przepukliny pępowinowej, osłonięcie przepukliny przez chirurga, bez zabiegu operacyjnego, w celu przekazania dziecka jak najszybciej pod opiekę hospicjum w warunkach domowych.

W tydzień po rozmowie, matka gorzej odczuwała ruchy płodu i została przyjęta do Kliniki Położnictwa, gdzie stwierdzono obumarcie płodu. Poród indukowano, drogami natury urodził się syn z masą ciała 1165 g, bez oznak życia.

Pacjentka wraz z mężem zostali objęci opieką psychologiczną.

Kilka tygodni później Mama napisała do obecnego przy porodzie neonatologa list, który wyraża to, co przeżyła. Oto jego fragmenty:

*[Położna] była z nami krótko (bo mój poród był krótki, jakieś 15 min) ale tak pięknie zaopiekowała się mną i moim Synkiem G. To Ona go chwyciła z dróg rodnych, powycierała i bardzo starannie ubrała. A potem z takim szacunkiem i miłością mi go podała mówiąc, ile waży i mierzy. Nie mogłam się oprzeć pokusie, by po wyjściu ze szpitala raz jeszcze do Niej nie przyjechać i wraz z mężem podziękować za wszystko*.

*Tak naprawdę, w IMID spotkaliśmy wiele Aniołów, takich jak Pani i Pani A. Nie raz zostałam przy tulona i wsparta dobrym słowem, troską, radą, pomocą. Ułatwiało mi to tę trudną sytuację. Nie wiedziałam, że są takie szpitale, jak Wasz i takie zespoły, jak Wasze. To były trudne chwile, ale miałam komfort bycia samej lub z mężem w samotności, intymności i godności. Nie musiałam z nikim rozmawiać, słuchać niczyich rozmów, mogłam do woli płakać. Mogłam długo żegnać się z G., przytulać Go, całować, kołysać*.

*[...] Razem z dziećmi machamy do Niego do Nieba, wysyłamy buziaki. Chodzimy na cmentarz, całujemy Jego grób, zbieramy kwiatki, czytamy Mu bajki. Gdy idę sama to z Nim rozmawiam*.

*Przestałam bać się śmierci, bo wiem, że wtedy odzyskam Go. Pozostał spokój i normalne życie. [...] Jakoś nie myślę o G. jak o chorym dziecku, bo nie miałam nawet chwili, by doświadczyć jego choroby. W brzuszku żył sobie jak każdy bobas, a poza brzuszkiem wyglądał pięknie jak moje pozostałe dwa Cuda. Był ciepły i pięknie pachniał*.

### Przykład 2

Pacjentka K. A-N: w trakcie diagnostyki prenatalnej, u płodu stwierdzono ogromny guz lity twarzy, szyi i klatki piersiowej przy jednoczesnym braku języka i krtani, potwierdzony 2-krotnym badaniem MRI.

Po wnikliwej analizie wady i dyskusji wewnątrz Zespołu, położnik, chirurg, neonatolog i anestezjolog spotkali się z pacjentką i jej mężem, by omówić postępowanie z matką i dzieckiem po urodzeniu. Rozmiar guza wymagał wykonania cięcia cesarskiego. Ze względu na lokalizację i charakter wady uniemożliwiające podjęcie czynności reanimacyjnych przewidywano, że dziecko po urodzeniu będzie żyło bardzo krótko, dlatego też szczegółowo omówiono, na czym będzie polegała opieka paliatywna. Rodzice poprosili o obecność taty dziecka w trakcie cesarskiego cięcia i o ochrzczenie dziecka zaraz po urodzeniu imieniem I. Poprosili także o możliwość przytulenia synka i spędzenia z nim możliwie długiego czasu w celu jego pożegnania. Wyrazili też chęć wykonania gipsowych odbić rączki i stopki.

Uzgodnione z rodzicami decyzje przedstawiono zespołowi położniczemu i neonatologicznemu.

Dziecko po urodzeniu żyło 20 min, spełniono wszystkie prośby rodziców. Matkę przewieziono na salę pooperacyjną w obrębie bloku operacyjnego, gdzie wraz z mężem, mogła pożegnać się z dzieckiem. Następnie zaproponowano rozmowę z Pracownikiem Socjalnym, który przedstawił rodzicom aspekty prawne związane z przygotowaniem pochówku dziecka.

Pacjentka wraz z mężem zostali objęci opieką psychologiczną.

### Przykład 3

Pacjentka S.S: w trakcie diagnostyki prenatalnej stwierdzono ogromną przepuklinę pępkową u płodu. W wykonanym w 21 tygodniu ciąży MRI potwierdzono wadę, stwierdzając jednocześnie pęknięcie przepukliny.

W trakcie dyskusji wewnątrz Zespołu zwrócono uwagę na rozległość wady. Konsultujący chirurg ocenił, że jest ona wadą nieoperacyjną. Po dyskusji członkowie Zespołu (położnik, chirurg, neonatolog) spotkali się z matką, by omówić sytuację jej dziecka. Rozmowa odbyła się w obecności matki pacjentki, mąż przebywał za granicą. Przedstawiono mamie 3 sytuacje, jakie mogą wystąpić w trakcie ciąży i po urodzeniu dziecka:

1) Ze względu na nieoperacyjny charakter wady, „ciężkie i nieodwracalne uszkodzenie płodu”, poinformowano matkę o przysługującym jej prawie wystąpienia o zgodę na wcześniejsze zakończenie ciąży (zgodnie z obowiązującą ustawą).

2) Możliwość wewnątrzmacicznego obumarcia płodu.

3) W przypadku kontynuacji ciąży, poród odbędzie się siłami natury, cięcie cesarskie jedynie w przypadku zagrożenia życia matki (np. gdy dojdzie do przedwczesnego oddzielenie się łożyska). Nie podejmowanie resuscytacji po urodzeniu z powodu nieoperacyjnej wady wrodzonej i natychmiastowe objęcie dziecka opieką paliatywną. Nie omawiano szczegółów opieki, oczekując na ostateczną decyzję matki, co do kontynuowania ciąży.

Po 3 dniach matka zgłosiła się do Kliniki Położnictwa w celu farmakologicznej indukcji porodu (22 tc). Drogami natury urodziła syna z masą ciała 240g bez oznak życia. Potwierdzono rozpoznaną prenatalnie wadę przepukliny pępowinowej z pęknięciem jej worka.

Pacjentka została objęta opieką psychologiczną.

## Wnioski i wskazówki praktyczne

### Z przedstawionych przykładów wynika, że

1) Pierwszym, a zarazem podstawowym zadaniem Zespołu Interdyscyplinarnego ds. Wad Płodu jest szczegółowe planowanie sposobu prowadzenia ciąży, jeśli matka wyraża chęć jej kontynuowania oraz wyznaczenie właściwego czasu i sposobu jej rozwiązania. Przy istniejących możliwościach terapii wewnątrzmacicznej ważne jest spersonalizowanie opieki nad ciężarną oraz płodem. Wszelkie decyzje muszą być poprzedzone rzetelną analizą. Położnik winien zebrać opinie możliwie najszerszego grona specjalistów oraz wystąpić o zwołanie posiedzenia Zespołu Interdyscyplinarnego. Na podstawie zebranych opinii formułuje on zalecenia w stosunku do matki i płodu, które następnie poddaje pod głosowanie. Gdy podjęcie decyzji nie wydaje się być oczywiste, należy umożliwić pacjentce przeprowadzenie dodatkowych badań w możliwie krótkim czasie.

2) Kolejnym zadaniem jest podjęcie refleksji nad sposobem postępowania z noworodkiem, w tym ewentualnie określenie sposobu prowadzenia opieki paliatywnej. Sytuacja taka zachodzi, gdy po rozpoznaniu ciężkiej wady wrodzonej dziecka, matka deklaruje chęć kontynuowania ciąży a członkowie zespołu na podstawie przeprowadzonych badań stwierdzają, że wdrażanie jakiegokolwiek leczenia (w tym podejmowanie działań resuscytacyjnych czy obciążanie dziecka zabiegiem chirurgicznym) nosiłoby znamiona uporczywej terapii. Sytuację taką spotykamy, gdy mamy do czynienia z tak zwaną „wadą letalną” lub zespołem wad o potencjalnie letalnym rokowaniu. Pod pojęciem „wady letalnej” często rozumie się ciężkie zaburzenia rozwojowe, które może prowadzić do zgonu wewnątrzmacicznego (20-40% przypadków) [6] lub niezależnie od zastosowanego leczenia do zgonu dziecka we wczesnym okresie noworodkowym, często bezpośrednio po urodzeniu. Jednak długość życia pojedynczego pacjenta nie jest możliwa do przewidzenia. Pozostaje zawsze element niewiadomej. Dlatego też w 2014 roku Wilkinson wraz ze współpracownikami [7] dokonali przeglądu piśmiennictwa, którego celem była ocena tego zjawiska. Okazało się, że niektóre z dzieci naznaczone tzw. „wadami letalnymi” żyły dużo dłużej, niż można by się tego spodziewać na podstawie powszechnych opinii. Na zwiększoną długość przeżycia miała wpływ zwłaszcza dojrzałość noworodka przy urodzeniu, zmniejszone nasilenie choroby wynikającej z wady (np. holoprozencefalia) oraz intensywność podejmowanego leczenia (w tym np. leczenia chirurgicznego). Wilkinson [7] proponuje wprowadzenie pewnej zmiany w istniejącej definicji wady letalnej, biorąc pod uwagę przede wszystkim czas zgonu płodu lub dziecka. Wadą letalną nazywa taką wadę, która powoduje z dużym prawdopodobieństwem zgon wewnątrzmaciczny lub zgon w okresie noworodkowym niezależnie od zastosowanego leczenia. Fakt ten zmienia perspektywę i powoduje, że lekarz w rozmowie z rodzicami powinien od początku wskazywać na dwie możliwe drogi naturalnego przebiegu choroby ich dziecka.

3) Po przeprowadzeniu dyskusji w ramach zespołu lekarz położnik i neonatolog winni przeprowadzić rozmowę z matką lub rodzicami i przedstawić im możliwe scenariusze dalszego prowadzenia lub wcześniejszego zakończenia ciąży. Rodzic musi zostać dokładnie poinformowany o stanie płodu i rokowaniach co do możliwości jego rozwoju. Przy czym informacja ta musi być podana w sposób jasny, odpowiedni, uczciwy i spójny:

**- jasny**: należy poświęcić cały niezbędny czas na wyjaśnienie zaistniałej sytuacji oraz wykazać się umiejętnością przełożenia żargonu medycznego na język zrozumiały dla rodziców. Dobrze byłoby uzyskać odpowiedź zwrotną ukazującą, na ile przekazana informacja została zrozumiana. Ów zwrot – to co rodzice zrozumieli – należy odnotować w karcie;

**– odpowiedni**: należy przekazać informacje dotyczące aktualnego stanu płodu, przedstawić diagnozę oraz rokowania, przewidywane scenariusze postępowania i ryzyko z nimi związane, łącznie z możliwością zgonu wewnątrzmacicznego lub śmierci noworodka zaraz po urodzeniu, jeżeli takie ryzyko istnieje. Należy przygotować rodziców do przyjęcia takiej informacji, by uniknąć nadmiernego wstrząsu przy i tak osłabionej równowadze psychicznej. Owa osłabiona równowaga nie może jednak stanowić powodu do ukrywania lub przemilczania pewnych istotnych informacji o stanie płodu i przewidywanych konsekwencjach. Nie należy przy tym podawać rodzicom zbyt wielu szczegółów medycznych. Przekazywane informacje nie powinny ograniczać się do przedstawienia samej patologii, ale winny one przedstawiać możliwe sposoby oraz konieczne do spełnienia warunki ewentualnego funkcjonowania dziecka. W przypadku konieczności wszczęcia postępowania paliatywnego należy zapewnić rodziców, że dziecko nie będzie odczuwało bólu ani głodu, nie będzie się dusiło i nie będzie mu zimno. Zapewniona będzie stała opieka lekarska oraz pielęgniarska. Informowaniu powinna jednocześnie towarzyszyć postawa uwagi na reakcje rodziców, wsłuchiwania się w ich obawy oraz uwzględnienie ich wątpliwości. Oczekiwania rodziców powinny zostać odnotowane w karcie;

**– uczciwy**: informacje muszą zawierać dane potwierdzone i pewne o aktualnym stanie płodu nawet, kiedy są one niekorzystne. Należy też uwzględnić element niepewności nieodłącznie związany z praktyką medyczną. Ważne może być przedstawienie dotychczasowego stanu wiedzy medycznej dotyczącej omawianej patologii;

**– spójny**: przekaz lekarza prowadzącego ciążę i neonatologa powinien być spójny z pozostałymi informacjami podawanymi przez poszczególnych klinicystów. Wszystkie one winne być zharmonizowane. Wymaga to dobrej komunikacji między członkami zespołu diagnostycznego, położniczego i neonatologicznego. Przekaz winien być oparty o odnotowane w karcie opinie. Lekarz przedstawiający swoją opinię winien na początku spytać pacjentkę: „Co Pani (Państwo) już wie na temat prowadzonej ciąży?”

Podczas rozmowy, a czasami i kilku rozmów, zależnie od stwierdzanej patologii brane są pod uwagę i omawiane różne możliwości prowadzenia ciąży, w tym możliwość zgonu w/macicznego. Omawiane są też ewentualne sposoby prowadzenia opieki paliatywnej, podejmowanej bezpośrednio na Sali porodowej lub leczenia paliatywnego, a jeżeli noworodek

przeżyje na Oddziale Intensywnej Terapii. Lekarz winien zebrać opinie rodziców, co do przedstawionych scenariuszy. Przy czym należy pozostawić rodzicom dziecka prawo do namysłu oraz prawo do decydowania o wcześniejszym zakończeniu ciąży zgodnie z obowiązującymi przepisami prawnymi lub jej kontynuowania i objęciu dziecka opieką paliatywną po urodzeniu. Lekarz nie może wyręczać rodziców w tym procesie decyzyjnym. W przypadku zgonu wewnątrzmacicznego lub śmierci dziecka bezpośrednio po urodzeniu diagnoza winna być potwierdzona, jeśli to możliwe, w badaniu autopsyjnym.

W przypadku kontynuowania ciąży płodu naznaczonego wadą letalną i konieczności wszczęcia postępowania paliatywnego już na sali porodowej w rekomendacjach dotyczących noworodka należy podkreślić bezwzględną potrzebę wyeliminowania bólu i drgawek u noworodka. Należy stale kontrolować nasilenie bólu, a w sytuacjach związanych z zakończeniem życia należy stosować nawet silne środki przeciwbólowe i przeciwdrgawkowe, o których wiemy, że ich podanie w dużych dawkach może doprowadzić do skrócenia życia pacjenta. Istotną rolę odgrywa tu intencja przepisującego leczenie przeciwbólowe, którą musi być zawsze skuteczne zapobieganie cierpieniu, nigdy zaś chęć skrócenia życia noworodka. Natychmiast po postawieniu rozpoznania ciężkiej wady płodu, należy zapewnić pomoc psychologa rodzicom przez cały czas trwania ciąży i po porodzie, tak długo jak będą tego potrzebowali.

Przedstawione tu zasady komunikowana zgodne są z oczekiwaniami rodziców, które zostały opracowane przez Stowarzyszenie Rodziców SPAMA *(Soins Palliatifs et Accompagnement en Maternité)* [8]:

– rodzice oczekują od lekarzy przede wszystkim wsparcia emocjonalnego w momencie przekazania diagnozy oraz cierpliwości w oczekiwaniu na podjęcie przez nich decyzji (okres płodowy) lub wyrażenia stanowiska i oczekiwań (po urodzeniu się dziecka);
– ważnym elementem jest rzetelne przedstawienie sytuacji klinicznej ich dziecka i sposobu prowadzenia opieki paliatywnej;
– powinno się umożliwić kontakt z noworodkiem, nawet na krótką chwilę tak, by matka i ojciec mogli poczuć się rodzicami;
– należy podjąć starania o umożliwienie obecności przy noworodku również pozostałym członkom rodziny;
– w końcu: towarzyszenie, również emocjonalne, rodzicom w pierwszych dniach żałoby ma dla nich fundamentalne znaczenie.

4) Po zakończonych rozmowach z rodzicami zakładana jest Karta, w której odnotowywane są wszystkie

dane kliniczne (w tym wyniki badań obrazowych, genetycznych itp.), streszczana jest rozmowa z rodzicami i odnotowane są wszystkie zgłaszane przez nich prośby. Karta przekazywana jest następnie zarówno zespołowi położniczemu, jak i stomatologicznemu, by niezależnie od momentu porodu, cały zespół medyczny (w tym pielęgniarki i położne) był poinformowany o podjętych decyzjach przekonsultowanych z rodzicami.

Odnotowywana jest również decyzja z posiedzenia Zespołu wraz z jej uzasadnieniem, a następnie przekazywana jest rodzicom przez neonatologa.

5) Ważne jest zaplanowanie sposobu weryfikacji rozpoznania prenatalnego zaraz po urodzeniu dziecka. Istotne znaczenie odgrywa tu zabezpieczenie materiału diagnostycznego, wykonanie dokumentacji fotograficznej, radiologicznej oraz szczegółowego opisu patologii. Postępowanie takie jest niezbędne dla zapewnienia prawidłowego poradnictwa i oceny ryzyka powtórzenia podobnej patologii u kolejnego potomstwa.

Zespół Interdyscyplinarny spełnia więc rolę *phronimosa*, mędrca obdarzonego ogromnym doświadczeniem i mądrością praktyczną, dzięki której może on wskazać właściwy teren intensywnej troski dla rodziców, stających często przed wyborem tragicznym, w którym równorzędne normy ścierają się ze sobą i nie ma takiej, która mogłaby rozstrzygnąć spór. Mądrość praktyczna gwarantuje poszanowanie norm moralnych przy jednoczesnym wyznaczeniu granic ich obowiązywalności. Zespół Interdyscyplinarny spełnia ważną rolę w podejmowaniu często trudnych i kontrowersyjnych decyzji, gwarantuje, że odpowiedzialność nie jest jednoosobowa, ale rozłożona na wszystkich członków zespołu, a doświadczenie zdobywane poprzez możliwość spojrzenia na daną sytuację z tak różnych stron ma ogromny wymiar edukacyjny. Dodatkowo rodzice mogą uzyskać informacje o problemach ich dziecka od wielu specjalistów i jednocześnie fachową pomoc od doświadczonych psychologów. Wszystko to sprawia, że życie rodziców i poszczególnych lekarzy jest mniej tragiczne i bardziej racjonalne.
